# Experimental and numerical evaluations on palm microwave heating for Red Palm Weevil pest control

**DOI:** 10.1038/srep45299

**Published:** 2017-03-31

**Authors:** Rita Massa, Gaetano Panariello, Daniele Pinchera, Fulvio Schettino, Emilio Caprio, Raffaele Griffo, Marco Donald Migliore

**Affiliations:** 1 Physics Department “Ettore Pancini”, University of Naples Federico II, Naples, Italy; 2 DIEI, University of Cassino and Southern Lazio, Cassino, Italy; 3 Department of Agricultural Sciences, University of Naples Federico II, Naples, Italy; 4 Plant Protection Service of Campania Region, Naples, Italy

## Abstract

The invasive Red Palm Weevil is the major pest of palms. Several control methods have been applied, however concern is raised regarding the treatments that can cause significant environmental pollution. In this context the use of microwaves is particularly attractive. Microwave heating applications are increasingly proposed in the management of a wide range of agricultural and wood pests, exploiting the thermal death induced in the insects that have a thermal tolerance lower than that of the host matrices. This paper describes research aiming to combat the Red Palm pest using microwave heating systems. An electromagnetic-thermal model was developed to better control the temperature profile inside the palm tissues. In this process both electromagnetic and thermal parameters are involved, the latter being particularly critical depending on plant physiology. Their evaluation was carried out by fitting experimental data and the thermal model with few free parameters. The results obtained by the simplified model well match with both that of a commercial software 3D model and measurements on treated *Phoenix canariensis* palms with a ring microwave applicator. This work confirms that microwave heating is a promising, eco-compatible solution to fight the spread of weevil.

The invasive Red Palm Weevil (RPW), *Rhynchophorus ferrugineus* Olivier (Coleoptera, Curculionidae) is one of the most destructive pests of palms in the world. It is widely distributed in Oceania, Asia, Africa and Europe^1^ and was recently found in the Caribbean^2^ and California^3^. RPW has been reported as a serious pest of coconut, oil palm, sago palm, date palm^1^ and in the Mediterranean Basin it is particularly destructive for *Phoenix canariensis* Hort. ex Chabaud. In September 2015, the pest was found also in plants of *Strelitzia nicolai* (Strelitziaceae) growing in Sicilia (IT)^4^. RPW pest has not only important economic consequences but also serious safety concerns. In fact, the breaking of the trunk, or toppling of the palm crown, due to the loss of the structural strength of the infested palm, can be a serious risk for people or things in nearby areas, particularly where these plants are used for ornamental purposes. Early detection of RPW-infested palms is crucial to avoid death of palms and is the key to the success of any Integrated Pest Management (IPM) strategy adopted to combat this pest. Based on the experience gained in Italy and other Mediterranean countries it is necessary to adopt preventive strategies that have primarily a protective character. If the palms are treated in the early stages of attack they can recover after treatments. The rehabilitation of the plant is based on the elimination of all vital biological insect stages present on the foliage and on the jamb. The employed techniques, whose outcome is never guaranteed in advance, include chemical, biological and biotechnological actions. All of them are essentially based on the elimination of infestations when they are initial, limited and have not compromised the stability of the plants. The control strategies to RPW in recent years still rely on “preventive treatments” and “curative treatments” of infected palm trees, while there are no specific interventions in countries or large areas where the presence of the pest is not highlighted. In IPM approach^5^ “preventive measures” are implemented either in the production of healthy plants for planting or on asymptomatic plants located in buffer areas as well as in focus zones contiguous to infested plants and where the danger of new infestations is high. For chemical treatments a minimum of eight treatments per season (from March to November) are recommended. Insecticides sprayed to the foliage, or localized to the apex vegetative, or applied endotherapy should be carried out with commercial products authorized by the respective national ministries; this implicates a big difference, between the countries, of the adopted molecules for applications either on ornamental palms or date palms. In addition, particular care should be considered especially during treatments on ornamental plants located in urban areas, where a low environmental impact is required (localized operations to the foliage, low or very low pressure applications, use of pipeline fixed on the top of the stipe down to a height of about 2.5 m.). Another strategy is pruning, a gradual removal of infested tissues aiming for complete insect removal (adults, larvae, pupae and eggs). The elimination of infected tissues must not undermine the vegetative apex on which new growth depends and should be recommended only in winter when the flight of adults is limited or absent. In fact wounds, such as those from pruning, emit volatiles that attract adult RPW, and thus pruning can increase the likelihood of a new infestation. Pruning of green leaves in the period of insect flight, even if associated with an insecticide treatment, is not sufficient to guarantee as the insecticide persistence is definitely less effective than the attractiveness of the cuts. Biological control based on entomopathogenic nematodes (EPNS) (*Heterorhabditis* and *Steinernema* species) despite being widely effective in the laboratory^6^ gave uncertain results in the field^7^, while mass trapping is generally only allowed under direct supervision of expert technicians. A trap set in an uninfested area can both easily lead to infestation by RPW responding to the attractive plumes coming from the trap, and it can greatly increase the incidence of weevils in an area if neighboring palms are not adequately protected. Studies were also conducted on entomopathogenic organisms associated with traps “attract, infect and release” to ensure the transition/detention of adults on substrate inoculated with indigenous strains of *Beauveria bassiana*. In this context, there is a considerable interest toward solutions able to control the pest with a minimum impact on the environment. A technology that could meet these apparently conflicting demands is based on microwave heating^8^. The basic idea is very simple: increasing the temperature of the insect until it dies. In general, the use of high temperature for insect pest control is based on the knowledge that insects have a limited physiological capacity to regulate their body temperature, resulting in a diverse number of adverse biochemical changes. Such an approach has been successfully applied to insect control in food, as grain and fruit^9^,^10^. Moreover phytosanitary treatment of wood^11^ by dielectric heating was recently formally approved by the Commission on Phytosanitary Measures of the International Plant Protection Convention (IPPC-FAO) as the first accepted alternative treatment to methyl bromide and conventional heating^12^. When exposed to radiofrequencies/microwaves the first reaction of insects is an attempt to escape; this is followed by motor coordination, stiffening, immobility and, after a certain time interval, death. Differences in susceptibility were also found between development stages within species. In general, the adult stages were more susceptible to control by radiation treatment than the immature stages^9^. Physiological examinations after exposures demonstrated behavioral and/or physical changes to insects even though at low intensity (less than 2 W/kg)^13^,^14^. With reference to RPW pest control, this approach, proposed by several authors^8^,^15^,^16^,^17^, has never been studied with respect to living plants. In fact reaching the RPW lethal temperature without damaging the palm is not straightforward. In particular, RPW attack typically occurs on the upper part of the trunk from the collar region near the crown, and the many life-stages (ovideposition, pupal development) occur in the region of the trunk near the surface where eggs, neonate larvae, cocoons and adults can be found while grubs are deeper inside. Microwave treatment basically works by increasing the temperature above the lethal value for RPW. Previous results regarding the thermal death kinetic showed that the adult insects are much more sensitive to heat than the larger larvae with 20 min at 50 °C and only 4 min at 80 °C causing adult death. Lethal time for the larvae varies with weight and the most resistant were those weighting between 4 and 6 g (30 min at 50 °C). The smallest larvae had a sensitivity similar to adults^8^. Once we demonstrated the feasibility^8^ and effectiveness^18^ of the treatment, we faced the problem of proposing a heating protocol using both simulations and experiments carried out in controlled conditions. To this end we developed a 1-D radial model. The validity of this simplified but straightforward approach was confirmed by comparing the results with both simulations, obtained with a 3D multiphysics electromagnetic-thermal simulator (Ansys), and measurements of the temperature distribution on palms treated with a ring microwave applicator. In this way a protocol can be suggested for unskilled operators once parameters such as dimensions of the palm, ambient temperature, thickness of the annulus of the palm section to be treated, are introduced.

## The Numerical Model

Broadly speaking, an analysis of the “*in vivo*” heating process of palms is extremely complex, since it involves heat transfer by movement of matter and changes of states. Such a complete model requires a large number of parameters that are nonlinear functions of temperature and water content, and whose values are not available in literature. In order to obtain affordable simulations, we can assume that during irradiation the evaporating water is rapidly substituted by other water drained by the plant. This assumption greatly simplifies the model. As a further approximation, the effect of the variation of the thermal and electromagnetic parameters, when the temperature increases, is neglected. With these two approximations the thermal and electromagnetic models are uncoupled, so we can first evaluate the RF dissipation in palm, and then perform a thermal simulation. We assume a cylindrically symmetric illumination around the trunk of the palm. Moreover longitudinal invariance is assumed, so that all physical quantities in both thermal and electromagnetic models only depend on the radial coordinate and time, and the analysis can be easily carried out by means of an FDTD approach^19^. On the basis of these assumptions the heat transfer equation is:


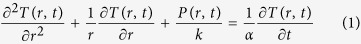


where *T* = *T(r, t*) [K] is the temperature profile as a function of the radial coordinate *r* [m] and time *t* [s], *P(r, t*) [W/m^3^] is the power loss density, *k* [W/(mK)] is the thermal conductivity of the material, *α* = *k*/(*c*_*p*_*ρ*) [m^2^/s] is the thermal diffusivity of the material, *ρ* [Kg/m^3^] is the mass density of the material, and *c*_*p*_ [J/Kg] is the specific heat capacity of the material. On the surface of the palm (*r* = *a*, wherein *a* is the radius of the palm) the effect of convection is modeled imposing the boundary conditions:


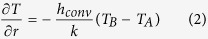


where *h*_*conv*_ [W/*m*^2^K] is the convection coefficient, *T*_*B*_ [K] is the surface temperature, and *T*_*A*_ [K] is the environment temperature. In order to use the FDTD approach, we consider a uniform sampling step for both *r* and *t*, obtaining the following finite difference equation


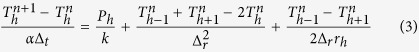


where *h* ∈ [1, *N*_*r*_] is the *h*-th radial position, 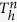 is the temperature in the *h*-th sampling radial position and at the *n*-th time instant, and *P*_*h*_ is the dissipated power density functions in the *h*-th sampling position. Equation 2 can be added in the system of equations by introducing the virtual temperature 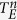 [K]^19^:


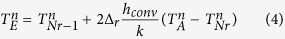


where 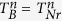, in order to calculate the discretized partial derivatives (3) also for the point *h* = *N*_*r*_ (i.e. on the surface):


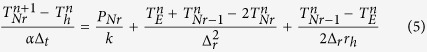


Electromagnetic characterization of the materials involved in the research was carried out in a previous work^20^ using the truncated coaxial cable technique and parameters at 2.45 GHz are reported in Table 1. On the other hand, the thermal parameters of wood, according to the results already presented in literature, can vary in relatively wide ranges. As an example, it is reported that specific heat and thermal conductivity of wood strongly depend on the moisture content^21^, and most of the known equations are not valid for a moisture content greater than 25% (which is the case of a living plant). Furthermore, it is not possible to characterize the thermal properties of the tissue of a living plant using the same approach that could be followed for a slab of lumber, since in the living plant the normal circulation of fluids for capillarity effect modifies the thermal behavior of the plant. For these reasons, the thermal conductivity and the specific heat capacity of the palm tissue were estimated matching the experimental data with the data obtained using the 1-D radial model of the heating process described in this section.

**Figure 1:**
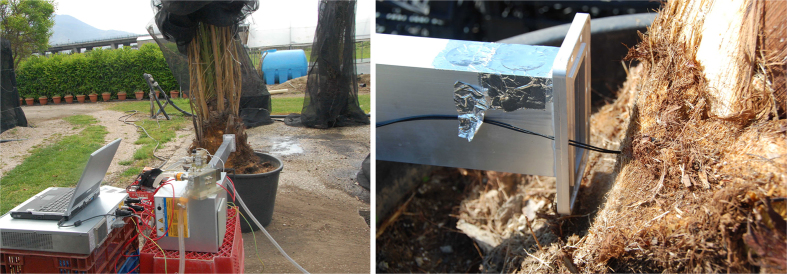
Experimental set-up to estimate the thermal parameters; (**a**) the palm is radiated by a WR340 waveguide connected to a microwave applicator; the incident power is measured by a circulator connected to a power meter; (**b**) the temperature in two points inside the palm is measured using an optical fiber thermometer.

**Figure 2:**
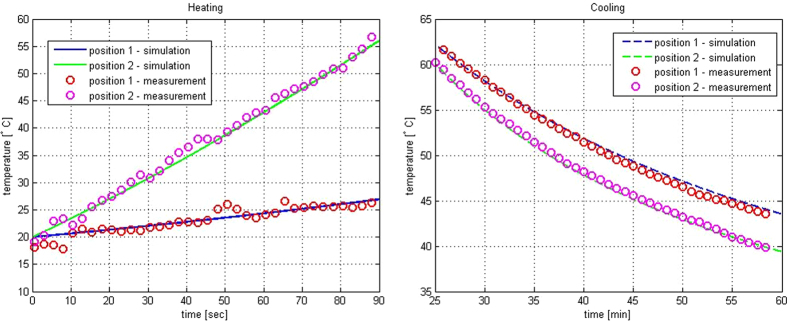
Comparison of the calculated and measured time behavior of the temperature during heating (left) and cooling (right).

**Figure 3:**
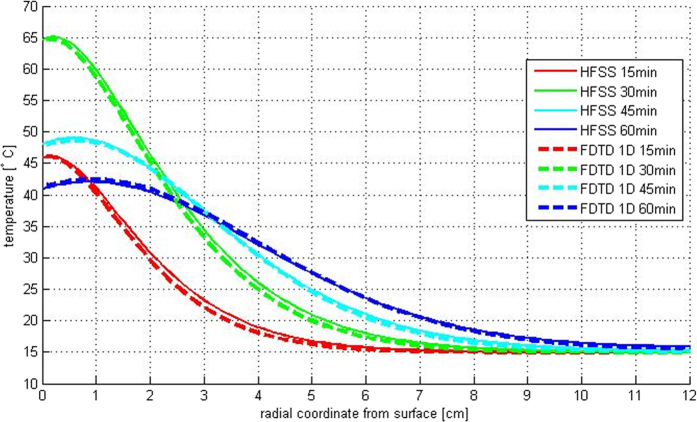
Validation of the custom FDTD thermal model by comparison of 1D code and HFSS 3D simulations showing the heating (red and green curves) and cooling (blue and cyan curves) process of a healthy palm (24 cm diameter, 40 cm height) microwave treated for 30 min and monitored for 60 min.

The experimental set-up consisted in a 2.45 GHz high power microwave source (Alter SN840 model TMA20) connected through a circulator and a directional coupler to a WR340 waveguide placed in front of the palm to be heated (see Fig. 1a). The incident power (1 kW) was measured by a power meter connected to the directional coupler, and two fiber optic probes, connected to the thermometer (Luxtron I652, accuracy ±0.5 °C), were placed at two different depths inside the palm (Fig. 1b) allowing accurate temperature measurements in two points along the radius of the palm (2 cm and 4 cm below the surface) during both the heating and the cooling process. In Fig. 2 the temperature behavior during heating and cooling phase respectively is shown. In order to find *k* and *c*_*p*_ the heating and cooling processes were simulated. The matching of the experimental and numerical curves was obtained using a least squares code in Matlab^®^. It must be noted that strictly speaking the parameters evaluated with this procedure are not the conductivity and the heat capacity of the palm tissue, but only equivalent conductivity and heat capacity able to simulate the heating and the cooling process, and could include the effect of a number of processes such as change of conductivity due to some slow water diffusion in the palm tissue. Finally, in order to validate the code described above, we compared the results obtained with the 1D code with the ones obtained for a 3D model simulated by a multiphysics full-wave commercial software (Ansys HFSS together with Ansys Thermal). The results confirmed the accuracy of the 1D code. As an example, in Fig. 3 we show the temperature profile curves for an ideal healthy palm of 12 cm radius and 40 cm height, uniformly heated for 30 minutes with a power source of 2 kW at 2.45 GHz, and left cooling for a further 30 minutes. The list of electromagnetic and thermal parameters used for the comparison is provided in Table 1, some of which are measured as described above. The good agreement of the results confirms the assumption that, due to the low thermal diffusion, the longitudinal dimension can be neglected, reducing the calculation time (HFSS simulation took more than one hour with a maximum edge length of the tetrahedral mesh of about 30 mm, whilst the solution using 1D code was almost instantaneous) and the complexity of the software that can be managed by an unskilled operator.

### Simulation of the microwave heating

**Figure 4:**
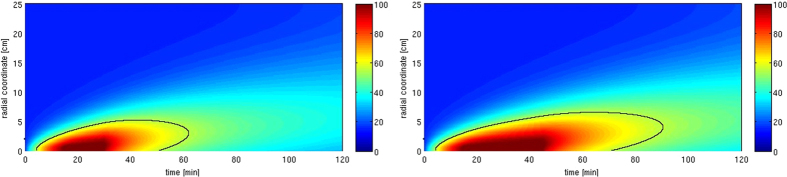
Simulation of the microwave heating of a 50 cm diameter palm for 30 (**a**) and 45 (**b**) minutes; the black curve indicates a temperature of 55 °C.

Once we have an accurate model for the living palm, we can correctly simulate the microwave heating process in order to establish which is the best heating protocol (i.e. the combination of RF power and exposure duration), in order to identify the duration of the microwave treatment to reach a desired temperature in the palm. In particular, the analysis undertaken in ref. 8 showed that a temperature of 55 °C for 30 min assures a probability of mortality higher than 90%. Accordingly, the aim of the simulation is to identify the thickness of the annulus of the palm section whose temperature is not lower than 55 °C in order to obtain a correct treatment. In this respect it must be noted that when the infestation has reached the inner part of the plant, it becomes useless and dangerous to treat the palm since the trunk has lost its mechanical strength. So, the treatment is particularly interesting in the early stages of infestation, when overall the outer part of the plant (whose thickness depends on the stage) has been infested. As an example we consider a palm (50 cm diameter), with an early stage infestation, treated with a ring applicator, having overall power of 12 kW on a vertical range of 40 cm and a microwave power efficiency of 50%, for 30 or 45 minutes. The parameters for the simulations are taken from Table 1. In Fig. 4 the temperature as a function of time in different radial positions inside the palm is shown. We can see that in the first case the lethal temperature of the RPW (*T*_*D*_ = 55 °C, black curve) is reached up to an abscissa of 5.2 cm below the surface (i.e. about 37% of the area of the section of the palm), while the temperature of the core of the palm (the inner 20 cm diameter core) is always below 27 °C. When the palm is illuminated for 45 minutes the plot shows that the RPW lethal temperature is reached up to an abscissa of 5.6 cm below the surface (i.e. about 40% of the area of the section of the palm), and the temperature of the core of the palm is always below 30 °C. The results show that,with the adopted conditions, the microwave heating regards only the outer section, while the core of the palm remains quite cool. This is a relevant feature, since it matches two important requirements: the layers involved in the process are the regions where overall both the early stage and the pupal development of the insects occur (it is worth noting that if the pest reaches the inner tissues, the palm irremediably dies and any treatment is useless); while keeping the core of the palm at lower temperature guarantees that microwaves do not affect the health of the plant.

### Validation of the model: experimental results with a ring applicator

**Figure 5:**
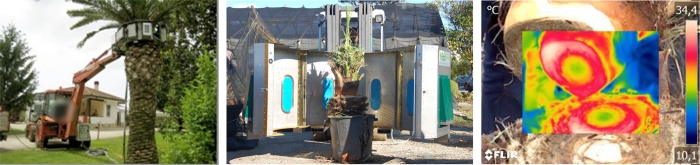
The ring applicator; (**a**) Ecopalm Ring patented by BIELLE surrounding the palm in closed position (by courtesy of BIELLE srl); (**b**) cut palm during just after treatment, the applicator is in the open position; (**c**) thermogram acquisition.

**Figure 6:**
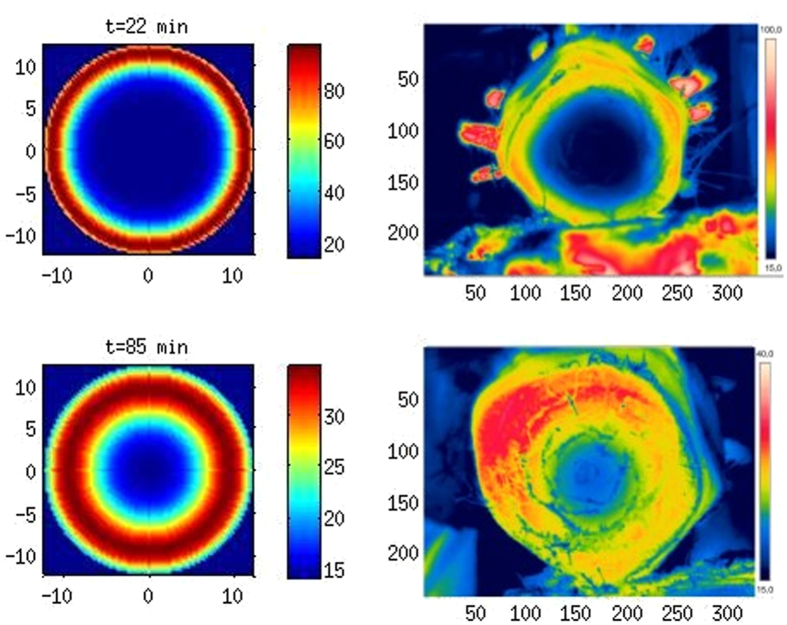
Temperature distribution in a section of a palm after microwave treatment: immediately after irradiation (above) and after about 1 hour from the end of the irradiation (below). The numerical results are on the left (axes in cm, temperature scale in Celsius degrees), the experimental results on the right (axes in mm, temperature scale in Celsius degrees).

**Figure 7:**
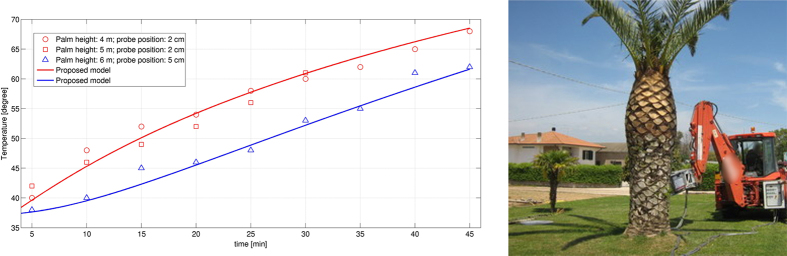
(**a**) Comparison of the time behavior of the temperature for three different palms with the proposed model; (**b**) one of the treated palms.

In order to check the results obtained by the numerical code we used a commercial ring microwave applicator (EcoPalm, patented by Bi.Elle s.r.l., showed in Fig. 5a and b). The applicator consists in 12 magnetrons (2.45 GHz, 1 kW nominal power) arranged in a ring that can be closed around the palm in order to surround a section of the trunk. Typically, it is located near the crown where usually RPW begins the attack. A set of measurements was carried out to evaluate the thermal distribution in a section of a treated palm. To this end a palm was carefully prepared by cutting away part of the leaves, then it was cut along a radial section (Fig. 5b) following a methodology similar to the one used in microwave hyperthermia to measure thermal distribution in phantom tissues^22^. The palm was radiated for 22 minutes, ambient temperature was 8 °C. As soon as the applicator was turned off, the upper part of the palm was lifted up and the thermal distribution was acquired using an IR thermocamera (Flir E6, accuracy ±2%). This operation required about 5 seconds. After acquiring the thermal distribution (Fig. 5c), the upper part of the palm was put back in its place, and lifted up periodically in order to measure the thermal distribution during the cooling process. As an example in Fig. 6 the temperature distribution 22 minutes and 85 minutes after switching off the microwave power are shown. It must be noted that since the palm is cut, the heat transfer by movement of matter is modified on the observed section. Hence, numerical simulation of the cut palm should require slightly different thermal parameters compared to a living palm due to the cut of the vascular system. However the comparison of the experimental and numerical results (Fig. 6) show an acceptable agreement. Similar results were observed by monitoring the temperature during microwave exposures of older (22–25 years) and bigger palms. In this case suitably shielded PT100 temperature probes were adopted, allowing to reach higher levels (4 m–6 m heights). In Fig. 7a the time behaviors of the temperature at 2 cm for two different palms and at 5 cm of another palm are shown and compared with our numerical model. In particular the palms were treated during spring/summer (24 °C–30 °C ambient temperature) with an 8 kW (nominal power) ring applicator (Fig. 7b). Generally speaking the experimental results prove that the heating induced by microwaves is approximately circularly symmetric on the section and that regards only the outer section, while the core of the palm remains relatively cool.

## Conclusions

In this paper we have discussed a simple numerical model able to simulate the temperature distribution inside a palm with reference to microwave heating for disinfestation of living plants attacked by RPW. To the best of our knowledge it is the first time that a microwave hyperthermia treatment of a plant is fully explored. A numerical code for the simulation of the electromagnetic and thermal problem has been obtained, as well as the parameters for its correct behavior. The results of the numerical model well match the general results obtained in an experimental investigation carried out on infested palms in a controlled environment. In particular we observed that it is difficult to reach a lethal temperature for RPW in the inner part of the trunk. In semi-field tests^18^ we observed a high percentage of dead insects (100% dead pupae in 3 over 4 palms, and 80% total) overall when they were in pupal period, i.e. blocked inside cocoons, that are typically located near the surface. It is worth noting that temperature higher than 27 °C can influence the longevity and fecundity of RPW^23^ and preliminary laboratory results indicate that the reproductive capacity of both male and female adults survived to a microwave exposure (5.4 W/cm^2^ for 5, 15, 30 sec) was reduced or removed depending on the treatment duration^24^, thus microwaves not only can be lethal for RPW but they could affect the development of new generation too. In conclusion, all these results indicate that high power microwave treatments are a very promising and eco-compatible solution for fighting the spread of RPW, which could be integrated in the IPM approach. As a matter of fact advantages of microwave and radiowave disinfestation include speed, efficiency, and the absence of toxic, hazardous or polluting residues. Moreover, insects are not likely to develop a resistance to radiation as they often do to chemical insecticides. The numerical model described in this paper will help to increase the efficiency of the microwave treatment allowing the estimation of the heat distribution inside the treated palm. The tool can be easily used by non-skilled operators for setting the parameters (duration, power, switch on-off etc.) that can better guarantee the efficacy of the treatment.

## Additional Information

**How to cite this article:** Massa, R. *et al*. Experimental and numerical evaluations on palm microwave heating for Red Palm Weevil pest control. *Sci. Rep.* **7**, 45299; doi: 10.1038/srep45299 (2017).

**Publisher's note:** Springer Nature remains neutral with regard to jurisdictional claims in published maps and institutional affiliations.

## Figures and Tables

**Table 1:** Electromagnetic and thermal parameters employed for the comparison of numerical and experimental evaluations.

*ε*′^20^	31.5	—
*ε*″^20^	11.5	—
k	1.5	*W*/*mK*
*h*_*conv*_	25	*W*/*m*^*2*^*K*
*ρ*^20^	844	*Kg*/*m*^3^
*c*_*p*_	3100	*J/Kg*
*T*_*A*_	25	°*C*
